# Cilostazol restores autophagy flux in bafilomycin A1-treated, cultured cortical astrocytes through lysosomal reacidification: roles of PKA, zinc and metallothionein 3

**DOI:** 10.1038/s41598-020-66292-3

**Published:** 2020-06-08

**Authors:** Ha Na Kim, Bo-Ra Seo, Hyunjin Kim, Jae-Young Koh

**Affiliations:** 1 Department of Neurology, University of Ulsan College of Medicine, Seoul, Korea; Department of Neurology, Asan Medical Center, University of Ulsan College of Medicine, Seoul, Korea; 2 Neural Injury Lab, Biomedical Research Center, Asan Institute for Life Sciences, Asan Medical Center, Seoul, Korea

**Keywords:** Cellular neuroscience, Molecular neuroscience

## Abstract

Cilostazol, a phosphodiesterase 3 inhibitor, reduces the amyloid-beta (Aβ) burden in mouse models of Alzheimer disease by as yet unidentified mechanisms. In the present study, we examined the possibility that cilostazol ameliorates lysosomal dysfunction. Astrocytes treated with bafilomycin A1 (BafA1) exhibited markedly reduced DND-189 and acridine orange (AO) fluorescence, indicating reduced lysosomal acidity. In both cases, BafA1-induced alkalization was reversed by addition of cilostazol, dibutyryl cAMP or forskolin. All three agents significantly increased free zinc levels in lysosomes, and addition of the zinc chelator TPEN abrogated lysosomal reacidification. These treatments did not raise free zinc levels or reverse BafA1-mediated lysosomal alkalization in metallothionein 3 (Mt3)-null astrocytes, indicating that the increases in zinc in astrocytes were derived mainly from Mt3. Lastly, in FITC-Aβ-treated astrocytes, cilostazol reversed lysosomal alkalization, increased cathepsin D activity, and reduced Aβ accumulation in astrocytes. Cilostazol also reduced mHtt aggregate formation in GFP-mHttQ74–expressing astrocytes. Collectively, our results present the novel finding that cAMP/PKA can overcome the v-ATPase blocking effect of BafA1 in a zinc- and Mt3-dependent manner.

## Introduction

Accumulation of abnormal protein aggregates is a common pathological finding in a variety of neurodegenerative disorders, including Alzheimer disease (AD) and Parkinson disease (PD)^1,2^. While initial studies focused on the mechanism by which protein aggregates are generated in a particular neurodegenerative disease, more recent studies have begun to ask questions relating to how formed protein aggregates are cleared in the central nervous system (CNS). This new direction may open up a broader path for finding potential treatments applicable to a number of protein aggregation-associated neurodegenerative diseases.

One of the most discussed mechanisms in this context is macroautophagy, or simply autophagy^3–6^. Whereas many misfolded proteins are degraded by the ubiquitin-proteasome system (UBS), large protein aggregates cannot be degraded by the UBS, and instead are cleared by autophagy. In this process, double-membrane–delimited autophagophores wrap around protein aggregates, resulting in the formation of autophagosomes, which then fuse with lysosomes. Digestion of the inner membrane of the autophagosome results in autolysosome formation, and lysosomal acidic hydrolases subsequently degrade protein aggregates. Hence, boosting autophagy may help catabolize protein aggregates that play pathogenic roles in neurodegenerative diseases. For instance, the autophagy-related protein beclin-1 is reported to be decreased in AD, which might lead to diminished autophagy^5,7–9^.

However, an increasing body of evidence indicates that instead of generalized defects in autophagy, lysosomal dysfunction that results in a decrease in autophagosome-lysosome fusion or autophagy arrest may be a more specific cause of the reduced autophagy flux^10–13^. More specifically, several studies have demonstrated that an alkaline shift in lysosomal pH may underlie these phenomena. For instance, presenilin mutations result in hypofunction of v-ATPase, a lysosomal proton pump^14–16^. Moreover, protein aggregates such as amyloid-beta (Aβ) and α-synuclein can shift the lysosome pH in a more alkaline direction. Hence, such a positive feedback loop might function as a vicious cycle that gradually increases the accumulation of protein aggregates. In fact, Nixon and colleagues have demonstrated that double-membrane–delimited autophagosomes containing Aβ accumulate in axons of AD brains^17–22^. If so, simply activating the upstream event, namely autophagosome formation, would not be very helpful in reducing Aβ accumulation in AD.

If abnormal lysosomal pH (i.e., alkalization) is the core pathologic change in these diseases, an ideal treatment is one that re-acidifies lysosomes. This might be accomplished in several ways. First, since it appears that v-ATPase activity may be reduced, for instance by presenilin mutations or Aβ aggregates, measures that increase v-ATPase activity might be helpful in these cases^23,24^. Although a direct v-ATPase activator is not known, studies have suggested that cAMP increases the assembly of v-ATPase in lysosomes^25–28^. A second strategy would be to seek measures that bypass v-ATPase routes and increase lysosomal proton levels via an alternative mechanism. For instance, lysosomal calcium extrusion via the non-selective cation channel, TRPML1 (transient receptor potential mucolipin 1), may help acidify lysosomes^29,30^. Interestingly, we reported that zinc ionophores that raise cytosolic and lysosomal free zinc levels can help acidify lysosomes in cells in which autophagy was arrested by chloroquine exposure^31^.

Cilostazol is a phosphodiesterase (PDE)-3 inhibitor that can increase intracellular cAMP levels^32–36^. It is approved for the treatment of intermittent claudication and prevention of ischemic heart attack and stroke^37–41^. Cilostazol was shown to prevent cerebral hypoperfusion-induced cognitive impairment and white matter damage^42–44^. It was also shown to be effective in decreasing the accumulation of Aβ in cellular and animal models of AD^45–47^. However, its precise mechanisms of action have not been elucidated. Because cAMP may affect lysosomal pH^48^, we examined the possibility that cilostazol’s effect on lysosomal pH may underlie this phenomenon. As a first approach, we examined whether cilostazol can reacidify lysosomes, even in the presence of the v-ATPase inhibitor BafA1, and whether changes in cytosolic/lysosomal free zinc levels are somehow involved in this process.

## Results

### Lysosomal reacidification by cilostazol or cAMP

**Figure 1:**
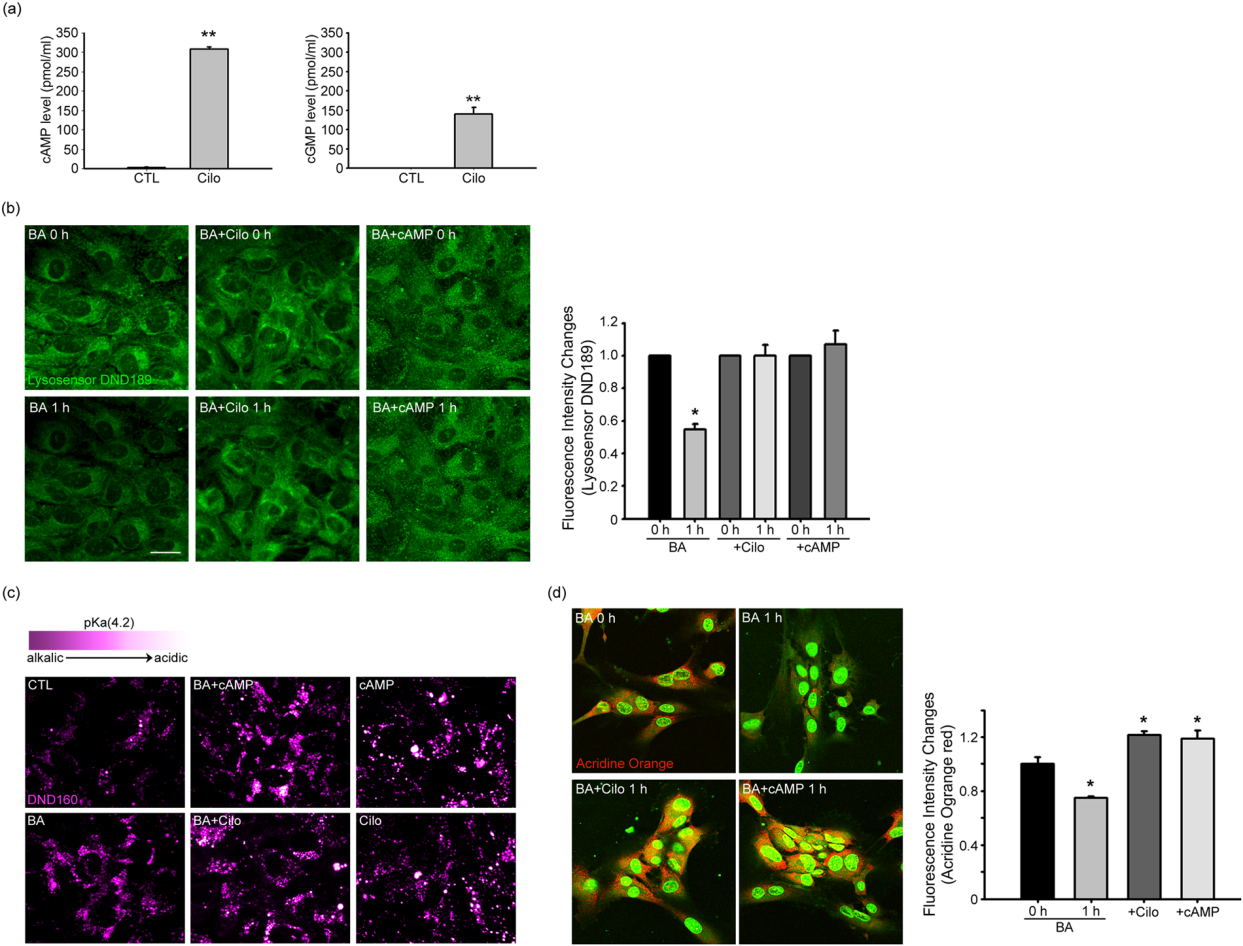
Lysosomes are re-acidified by cilostazol or cAMP in cultured cortical astrocytes. (**a**) Bars denote cAMP and cGMP levels (pmol/ml) in cultured astrocytes. A 1-hour treatment with 10 μM cilostazol increased cAMP and cGMP levels in astrocytes. Data are presented as means ± SEM (n = 6; **P < 0.01). (**b**) Fluorescence photomicrographs of cultured cortical astrocytes loaded with DND-189, before and after a 60-minute exposure to BafA1 (BA) alone, BafA1 plus cilostazol (BA + Cilo) or BafA1 plus cAMP (BA + cAMP). BafA1 substantially reduced DND-189 fluorescence via its v-ATPase–inhibiting effect, an action that was blocked by the addition of cilostazol (n = 6; Scale bar, 20 μm). Bars denote relative fluorescence intensity changes (0 h as 1, mean + SEM, n = 6) in these conditions. (* denote *P* < 0.05, Two-tailed Student’s t-test for 2 comparisons). (**c**) Fluorescence photomicrographs of cultured cortical astrocytes loaded with a ratiometric pH sensitive dye DND-160, before and after a 60-minute exposure to BafA1 (BA) alone, BafA1 plus cilostazol (BA + Cilo) or BafA1 plus cAMP (BA + cAMP). Colorimetric fluorescence images of astrocytes stained with the pH-sensitive lysosomal dye DND-160 (LysoSensor); purple denotes more alkaline and white more acidic pH. (Scale bar, 20 μm). (**d**) Fluorescence photomicrographs of cultured cortical astrocytes loaded with acridine orange (AO), another pH-sensitive fluorescent dye, and treated as in B. Again, BafA1 markedly reduced AO fluorescence in lysosomes, an effect that was almost completely reversed by the addition of cilostazol (BA + Cilo) or cAMP (BA + cAMP). Bars denote relative fluorescence intensity changes (0 h as 1, mean + SEM, n = 6) in these conditions. (* denote *P* < 0.05, Two-tailed Student’s t-test for 2 comparisons).

To test the effect of cilostazol in cultured astrocytes, we first measured changes in cAMP levels. Consistent with its potent effect as a PDE inhibitor, cilostazol (10 μM) treatment for 1 hour markedly increased the level of cAMP in astrocytes. Cilostazol also induced a concurrent increase in cGMP levels, albeit to a lesser degree than cAMP levels (Fig. 1a). This pattern is consistent with the relative non-selectivity of PDE3 being towards cAMP and cGMP hydrolysis. Next, to assess lysosomal pH changes, we loaded cultured cortical astrocytes with DND-189, a lysosomal-specific pH-sensitive fluorescent dye. In the resting state, lysosomes exhibited intense DND-189 fluorescence, reflecting the acidic pH of the normal lysosomal lumen (Fig. 1b). After a 60-minute exposure to 100 nM bafilomycinA1 (BafA1), a potent and selective inhibitor of the lysosomal proton pump, v-ATPase, DND-189 fluorescence in lysosomes was substantially dimmed, as expected, indicating an alkaline shift in lysosomal pH (Fig. 1b). Notably, addition of 10 μM cilostazol or 300 μM cAMP largely abrogated the BafA1-induced changes in lysosomal pH (Fig. 1b). To further confirm this effect, we turned to other pH-sensitive fluorescent dyes, acridine orange (AO) and Lysosensor Yellow/Blue DND-160 (DND-160). The ratiometric lysosomal pH indicator, DND-160, yielded results similar to DND-189 (changing from white to purple with increasing lysosomal pH) (Fig. 1c). Acridine orange (AO), which emits green fluorescence upon binding to nuclear DNA, emits orange fluorescence under acidic pH environments such as in lysosomes. Whereas BafA1 did not alter the green fluorescence of nuclear AO, it markedly reduced orange fluorescence in lysosomes. Again, addition of 10 μM cilostazol or 300 μM dibutyryl cAMP (cAMP analog) blocked the BafA1-induced loss of AO fluorescence in lysosomes (Fig. 1d). By comparison, the effect of cGMP was much less pronounced than that of cAMP (not shown). Taken together, these results indicate that increasing cAMP levels may be effective in restoring lysosomal acidity, even in the presence of the potent v-ATPase inhibitor BafA1.

### PKA may mediate the effects of cilostazol and cAMP

**Figure 2:**
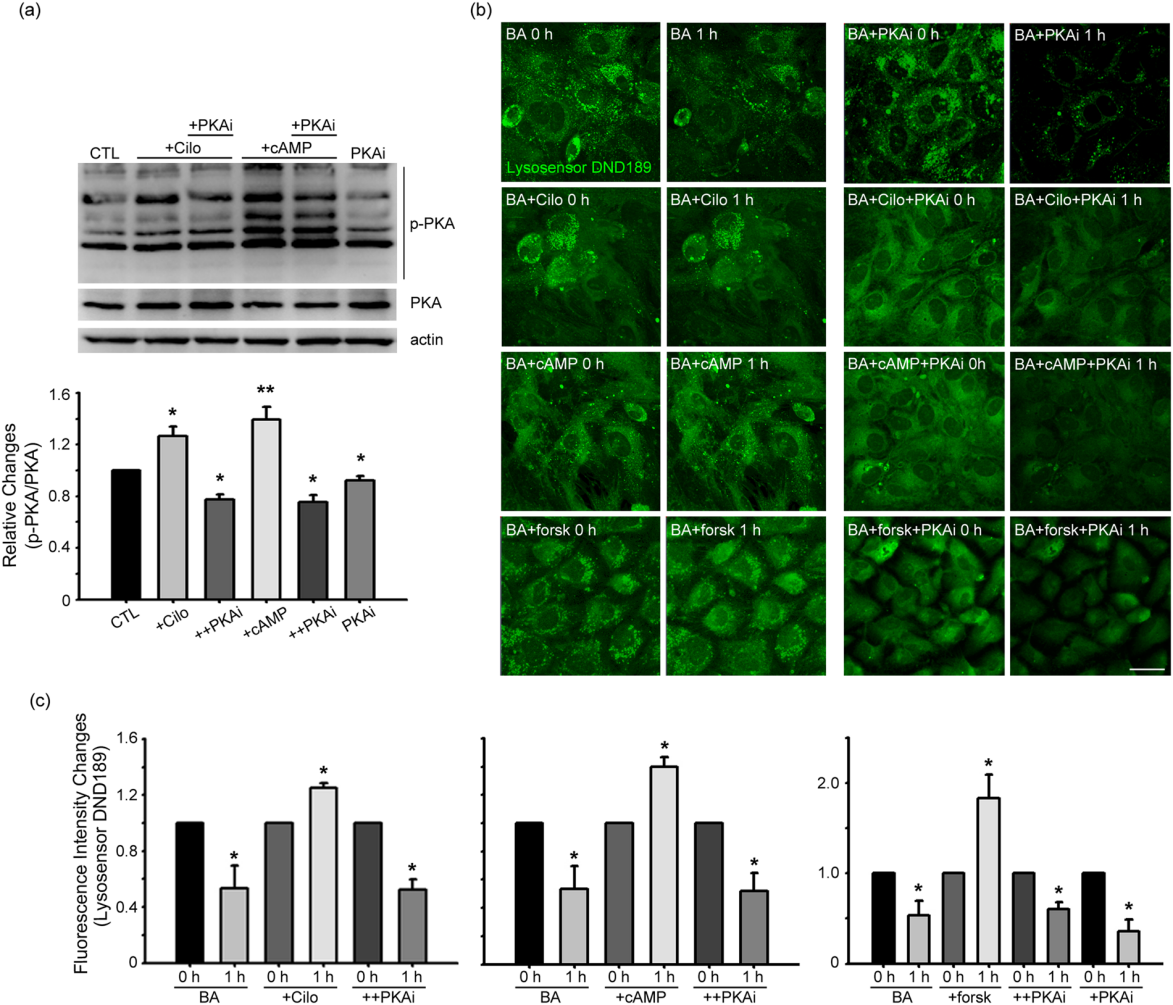
PKA may mediate the effects of cilostazol and cAMP. (**a**) Cilostazol and cAMP activate PKA in astrocytes. Western blot analysis of p-PKA in cells treated with 10 μM cilostazol (+Cilo) or 300 μM cAMP alone (+cAMP), or together with 10 μM H-89 (+PKAi), a PKA inhibitor, for 1 hour. H-89 reversed cilostazol- or cAMP-induced increases in p-PKA levels. Bars denote the ratio of p-PKA bands to corresponding total PKA bands. Data are presented as means ± SEM (n = 7; * denote *P* < 0.05, ** denote *P* < 0.01 compared with CTL, Cilo, cAMP or PKAi, Two-tailed Student’s t-test for 2 comparisons). (**b**) DND-189 fluorescence in astrocytes before (CTL) and after a 60-minute exposure to BafA1 (BA), BafA1 plus cilostazol (BA + Cilo), BafA1 plus cAMP (BA + cAMP) or BafA1 plus forskolin (BA + forsk), in the absence or presence of PKAi (BA + PKAi) (Scale bar, 20 μm). (**c**) Bars indicate relative changes in the fluorescence intensity. Values for individual bars were normalized to control values (mean ± SEM; n = 4; * denote *P* < 0.05 compared with BA, Cilo, cAMP, forsk or PKAi; Two-tailed Student’s t-test for 2 comparisons).

cAMP activates several cells signaling cascades, including the protein kinase A (PKA) pathway. Consistent with this, levels of phosphorylated PKA (p-PKA) in astrocytes were increased by treatment with cilostazol or cAMP (Fig. 2a). Accordingly, we examined the possibility that PKA mediates the effect of cilostazol/cAMP on lysosomal pH. To this end, we tested whether inhibiting PKA prevented cilostazol or cAMP from blocking the decrease in DND-189 fluorescence induced by BafA1. Consistent with a role for PKA activity, addition of the PKA inhibitor H-89 almost completely reversed the effects of cilostazol and cAMP, reducing DND-189 fluorescence to a level comparable to that in BafA1-treated astrocytes. Forskolin, a PKA activator, exerted the same effect as cilostazol and cAMP on lysosomal pH changes induced by BafA1 (Fig. 2b,c).

### Cilostazol and cAMP increase lysosomal free zinc levels

**Figure 3:**
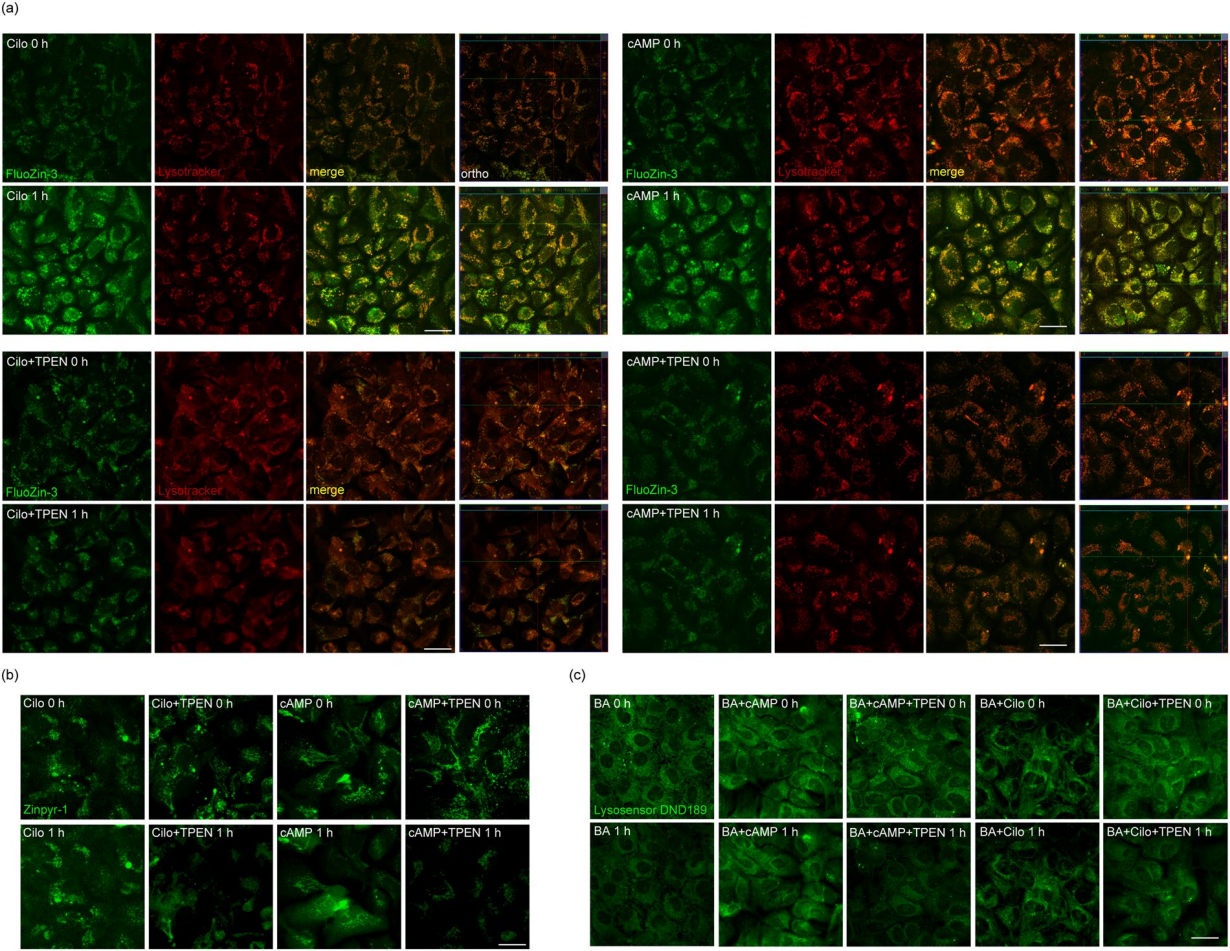
Lysosomal free zinc levels are increased by treatment with cilostazol or cAMP. (**a**) Changes in intracellular and lysosomal free zinc levels were visualized by loading astrocytes with both FluoZin3-AM and LysoTracker for 30 minutes, followed by a sham wash (CTL) or treatment for 60 minutes with 10 μM cilostazol (n = 5) or 300 μM cAMP (n = 4). LysoTracker-positive particles exhibited red fluorescence in resting astrocytes (CTL). Cilostazol and cAMP further increased free zinc levels, most of which colocalized with LysoTracker fluorescence (Scale bar, 20 μm). (**b**) Changes in intracellular and lysosomal free zinc levels were visualized by loading astrocytes with a zinc-specific fluorescent dye Zinpyr-1 for 30 minutes, followed by a sham wash (CTL) or treatment for 60 minutes with 10 μM cilostazol, or 300 μM cAMP (n = 3) (Scale bar, 20 μm). (**c**) DND-189 fluorescence in astrocytes, before and after exposure to BafA1 alone (BA), BafA1 plus cilostazol (BA + Cilo), or BafA1 plus cAMP (BA + cAMP). Further addition of TPEN completely blocked the lysosomal pH-reversing effects of cilostazol (BA + Cilo+TPEN) and cAMP (BA + cAMP+TPEN) (Scale bar, 20 μm).

Next, based on our previous results showing that lysosomal free zinc levels may affect lysosomal pH^49^, we examined whether treatment with cilostazol or cAMP altered free zinc levels in astrocytes. Astrocytes were loaded with FluoZin-3-AM and Zinpyr-1 (Fig. 3b), a zinc-sensitive fluorescent dye, and with LysoTracker and LAMP (Supplementary Fig. 5), which stains lysosomes. Then astrocytes were treated with 10 μM cilostazol or 300 μM cAMP for 1 hour. Both treatments substantially increased zinc fluorescence in a particulate pattern, an effect that was completely blocked by the membrane-permeant zinc chelator, TPEN (Fig. 3a,b). Merged images of zinc and lysosome signals showed that the majority of the increased zinc fluorescence was indeed localized in lysosomes (Fig. 3a). We then tested whether zinc had any role in cilostazol- or cAMP-mediated lysosomal reacidification in BafA1-exposure conditions. Lysosomal reacidification by cilostazol or cAMP in BafA1-treated astrocytes was completely blocked by the addition of TPEN, suggesting that zinc plays an essential role in this phenomenon (Fig. 3c).

### Mt3 may be the main source of zinc increases following cAMP treatment

**Figure 4:**
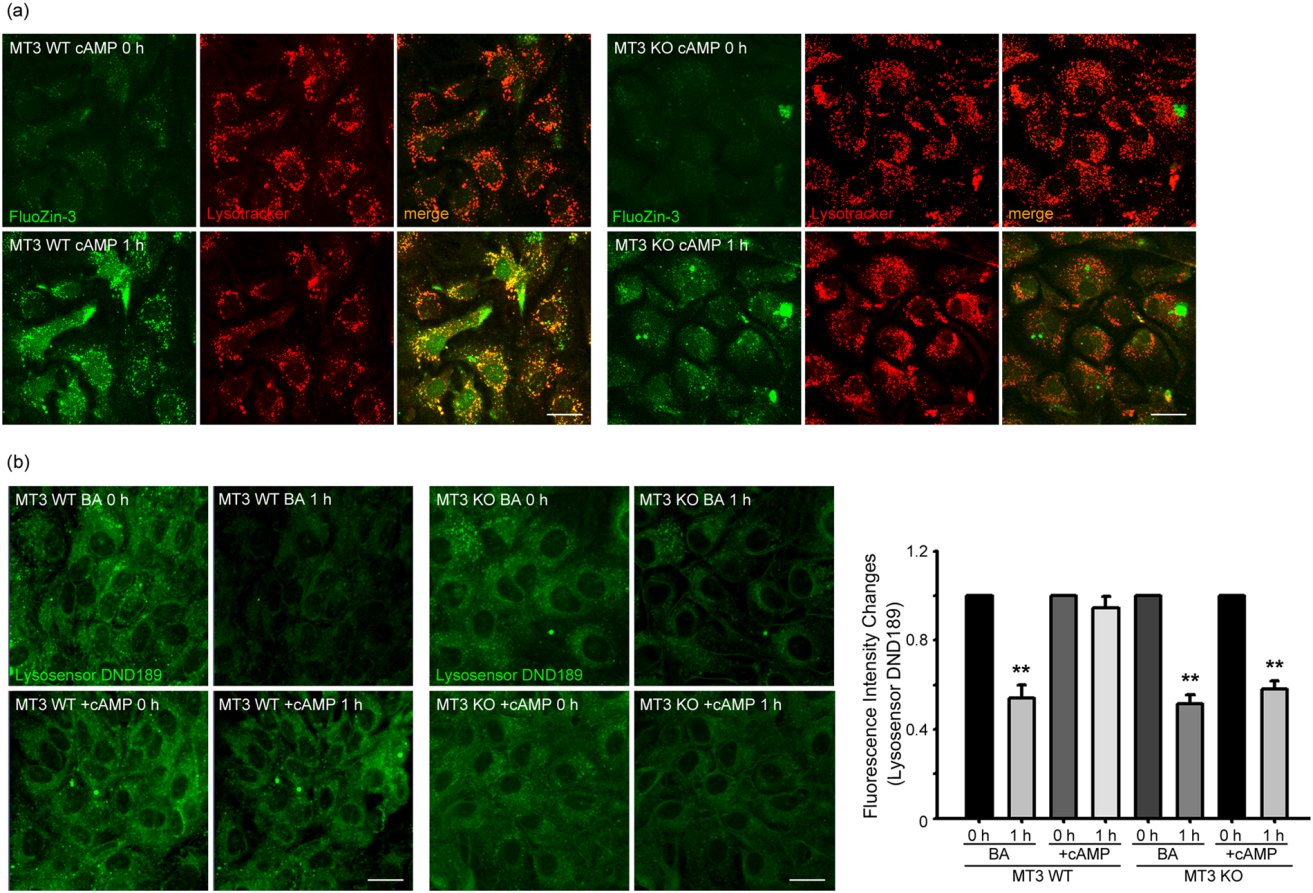
Mt3 may be the main source of zinc increases following cAMP treatment. (**a**) Mt3-WT (left) astrocytes before (MT3 WT cAMP 0 h) and 60 minutes (MT3 WT cAMP 1 h) after exposure to 300 μM cAMP. FluoZin3 and LysoTracker fluorescence microscopy showed increases in free zinc levels in the cytosol and lysosomes following cAMP exposure. A 60-minute exposure to cAMP failed to increase intracellular and lysosomal free zinc levels in Mt3-null astrocytes (right) (n = 4; Scale bar, 20 μm). (**b**) Fluorescence photomicrographs of DND-189-loaded Mt3-WT (left) and Mt3-null (right) astrocytes, before and after a 60-minute exposure to BafA1 alone or BafA1 plus cAMP (Scale bar, 20 μm). BafA1 markedly reduced DND-189 fluorescence in both cultures. However, addition of cAMP reversed the effect of BafA1 only in WT astrocytes. Bars indicate relative changes in the fluorescence intensity. Values for individual bars were normalized to control values (mean ± SEM; ** denote *P* < 0.01 compared with BA or cAMP; Two-tailed Student’s t-test for 2 comparisons).

Intracellular free zinc levels appear to be meticulously regulated by diverse mechanisms^50–52^. We previously showed that metallothionein 3 (Mt3), a brain-enriched form of metallothionein, is the main source of intracellular zinc released under conditions of oxidative or nitrative stress in cultured cortical astrocytes^53,54^. Moreover, Mt3 appears to modulate lysosomal pH and its functions. Hence, we examined whether Mt3 serves as a significant source of zinc for cilostazol and cAMP/PKA-induced increases in cytosolic and lysosomal free zinc levels. To this end, we cultured cortical astrocytes obtained from brains of Mt3-wild-type (WT) and Mt3-null newborn mice. Exposure to 300 μM cAMP markedly increased lysosomal free zinc levels in WT astrocytes, but had a much lesser effect in Mt3-null astrocytes (Fig. 4a), suggesting that cAMP-induced increases in zinc in astrocytes are dependent on the presence of Mt3. Next, we exposed DND-189-loaded Mt3-WT and -null astrocytes to BafA1 or BafA1 plus cAMP. In WT astrocytes, as previously shown, BafA1 markedly reduced DND-189 fluorescence, an effect that was largely reversed by addition of cAMP. However, in Mt3-null astrocytes, addition of cAMP failed to restore lysosomal acidity in the presence of BafA1 (Fig. 4b). Taken together, these results indicate that cAMP/PKA-mediated zinc release from Mt3 may play a key role in negating BafA1 effects on lysosomal pH.

### Cilostazol alleviates Aβ-induced disruption of lysosomal pH and activity in cultured cortical astrocytes

**Figure 5:**
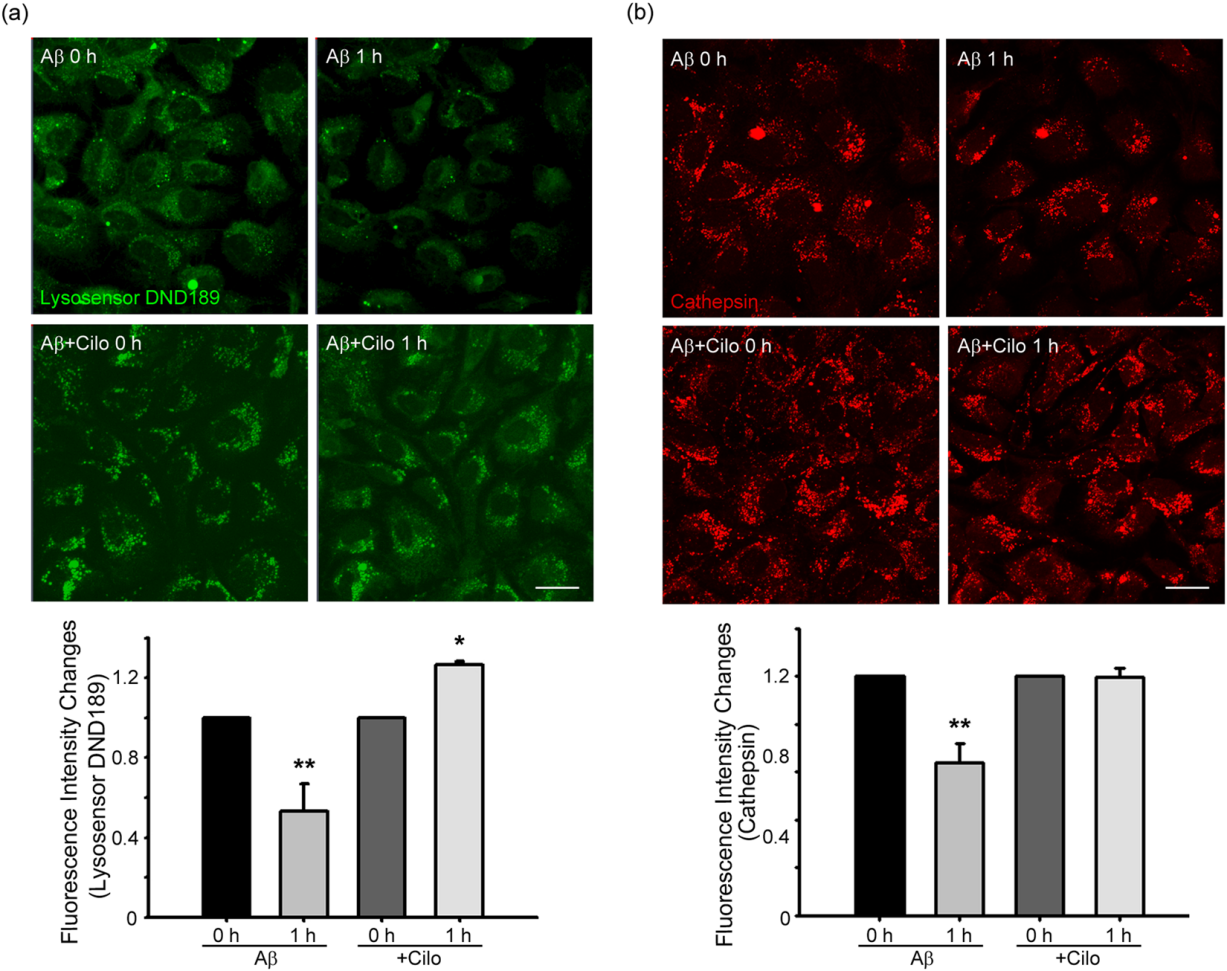
Cilostazol alleviates Aβ-mediated disruption of lysosomal pH and activity in cultured cortical astrocytes. (**a**) Aβ induces lysosomal alkalization. Astrocytes were loaded with Aβ_1–42_ for 60 minutes and examined for DND-189 fluorescence (Scale bar, 20 μm). Whereas Aβ exposure markedly reduced DND-189 fluorescence (alkalization), addition of cilostazol (Aβ + Cilo) almost completely blocked the Aβ effect on lysosomal pH. Bars indicate relative changes in the fluorescence intensity. Values for individual bars were normalized to control values (mean ± SEM; n = 4; * denote *P* < 0.05, ** denote *P* < 0.01 compared with Aβ or Cilo; Two-tailed Student’s t-test for 2 comparisons). (**b**) Aβ decreases the activity of cathepsin B, a member of the lysosomal cysteine protease family. Fluorescence photomicrographs of cathepsin B-loaded astrocytes, before and after a 60-minute exposure to Aβ alone (Aβ) or Aβ plus cilostazol (Aβ + Cilo) comparisons (Scale bar, 20 μm). Aβ markedly reduced cathepsin B fluorescence in astrocytes, an effect that was reversed by addition of cilostazol. Bars indicate relative changes in the fluorescence intensity. Values for individual bars were normalized to control values (mean ± SEM; n = 4; * denote *P* < 0.05 compared with Aβ or Cilo; Two-tailed Student’s t-test for 2 comparisons).

Next, we examined whether cilostazol exerts beneficial effects on Aβ-induced changes in astrocytes. Exposure of astrocytes to 1 μM Aβ for 1 hour markedly reduced DND-189 fluorescence intensity in lysosomes, indicating that Aβ treatment somehow interfered with lysosomal acidification^48^ (Fig. 5a). In contrast, in astrocytes exposed to Aβ plus 10 μM cilostazol, DND-189 fluorescence intensity remained unchanged (Fig. 5a). In addition to its effect on lysosomal acidity, Aβ exposure reduced the activity of cathepsin B in astrocytes, as assayed using Magic Red cathepsin B assays. Again, addition of cilostazol largely prevented the Aβ-induced reduction in cathepsin B activity (Fig. 5b).

### Cilostazol reduces accumulation of Aβ and huntingtin aggregates

**Figure 6:**
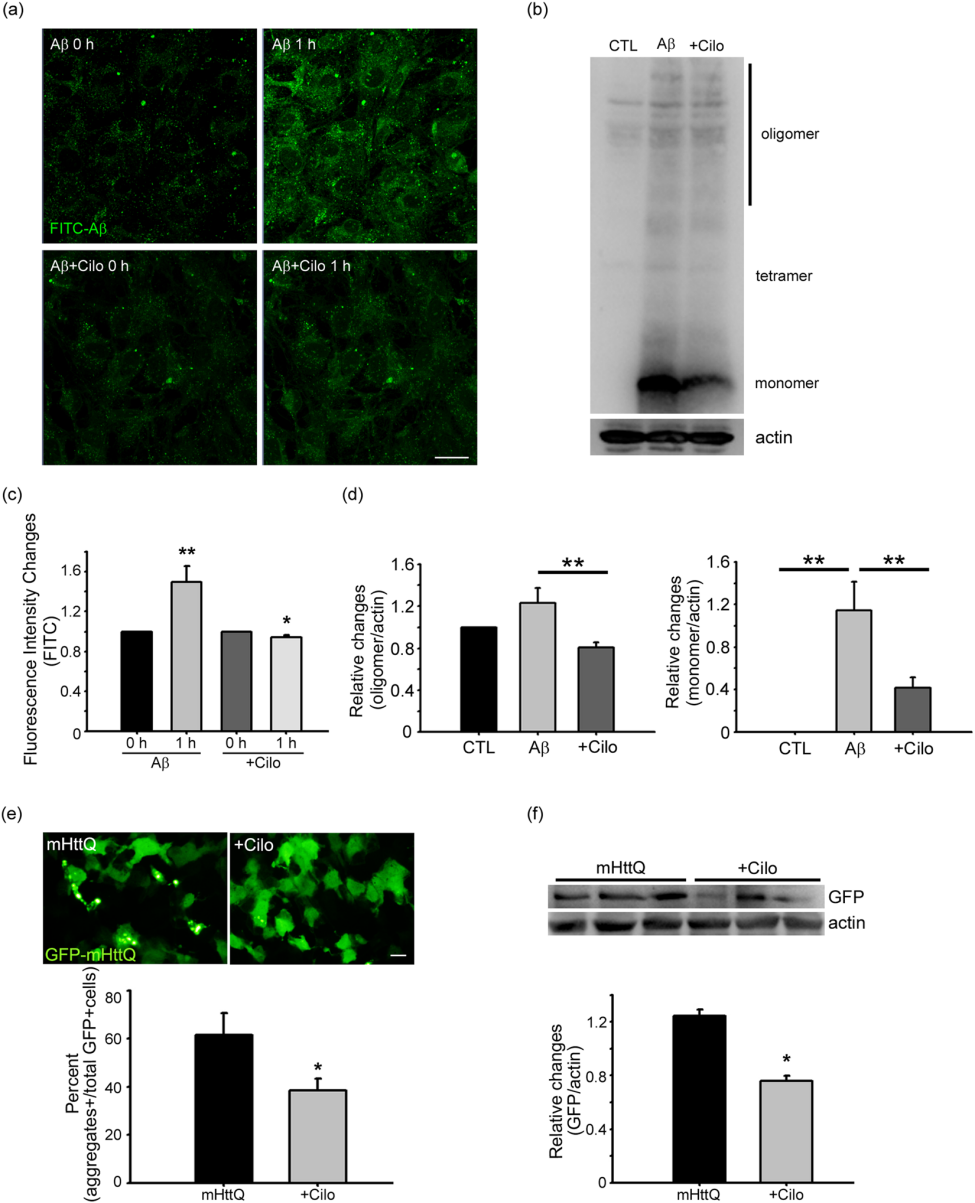
Cilostazol reduces accumulation of Aβ and huntingtin aggregates in astrocytes. (**a**,**c**) Astrocytes were exposed to FITC-Aβ for 1 hour. Fluorescence photomicrographs taken after washout show increased Aβ levels in astrocytes (Scale bar, 20 μm). Addition of cilostazol substantially reduced Aβ loads in astrocytes. Bars indicate relative changes in the fluorescence intensity. Values for individual bars were normalized to control values (mean ± SEM; n = 7; * denote *P* < 0.05, ** denote *P* < 0.01 compared with Aβ or Cilo; Two-tailed Student’s t-test for 2 comparisons). (**b**,**d**) Western blot analyses of lysates from cells that were sham-washed (CTL), or exposed for 24 hours to Aβ alone or Aβ plus cilostazol (+Cilo). Levels of Aβ monomers, tetramers, and oligomers were decreased by Aβ plus cilostazol compared with Aβ alone. Bars represent the ratio of 6E10 bands to corresponding β-actin bands (mean ± SEM; n = 6; ** denote *P* < 0.01 compared with CTL, Aβ or Cilo; Two-tailed Student’s t-test for 2 comparisons). (**e**) Cilostazol reduces mHttQ74 aggregation in astrocytes. Fluorescence photomicrographs of GFP-mHttQ74–transfected astrocytes. Six hours after transfection, astrocytes were treated with cilostazol (+Cilo) for 14 hours. White dots indicate GFP-positive aggregates. Bars denote the percentage of GFP-aggregate-positive cells in all GFP-positive cells (mean ± SEM; n = 5; * denote *P* < 0.05 compared with mHttQ or Cilo; Two-tailed Student’s t-test for 2 comparisons). Western blot analysis of GFP aggregates obtained from GFP-mHttQ74–overexpressing astrocytes following cilostazol treatment. Transfected cells were sham-washed (mHttQ) or treated with 10 μM cilostazol (+Cilo) for 14 hours. Bars represent the ratio of GFP bands to corresponding β-actin bands (n = 4; * denote *P* < 0.05 compared with mHttQ or Cilo; Two-tailed Student’s t-test for 2 comparisons).

Finally, we examined whether cilostazol reduces accumulation of the toxic protein aggregates, Aβ and huntingtin, in astrocytes. Aβ accumulation was measured in astrocytes loaded with FITC-Aβ. Cilostazol, added after washout, markedly reduced the amount of FITC-Aβ in astrocytes (Fig. 6a,c). Western blots also revealed that accumulation of both monomeric and oligomeric fractions of Aβ in astrocytes was significantly reduced by cilostazol (Fig. 6b,d). To measure huntingtin accumulation, we transfected astrocytes with GFP-tagged 74 polyQ-repeat mutant human huntingtin (GFP-mHttQ74). In these cells, formation of GFP-positive protein aggregates was detected beginning 14 hours after transfection. Compared with controls (GFP-mHttQ74 only), addition of 10 μM cilostazol (+Cilo) substantially reduced the accumulation of aggregates (Fig. 6e). Western blot analyses using an anti-GFP antibody confirmed these findings (Fig. 6f).

## Discussion

Abnormalities in lysosomal function are increasingly recognized as significant and common mechanisms that contribute to the pathogenesis of diverse neurodegenerative disorders associated with abnormal protein aggregates^55–57^. More specifically, inadequate acidification of the lysosomal lumen is a common change, one that not only decreases the activity of degradative enzymes in lysosomes, but also impedes the fusion of lysosomes with autophagosomes and endosomes^11,58,59^. Notably, a growing body of evidence suggests that abnormal protein aggregates per se may be a cause of lysosomal alkalization, raising the intriguing possibility of a vicious cycle of amplification. For instance, in AD, presenilin mutations as well as Aβ oligomers cause lysosomal alkalization^48^. α-Synuclein also causes the same effect in PD^60,61^. Even in prion diseases, the alternatively folded prion protein, PrPSc, induces lysosomal alkalization^62^. Considering that lysosomes are the major, and perhaps only, path for the degradation of large-size protein aggregates, such changes could play a crucial role in disease progression. Therefore, normalization or reacidification of lysosomal pH should be considered a potential therapeutic strategy for these disorders.

The results presented here showed that cilostazol, a clinically used PDE3 inhibitor, successfully restored lysosomal pH in the presence of BafA1, a potent inhibitor of v-ATPase, which functions as the main proton pump for lysosomes. This effect is likely mediated by increases in cAMP levels and subsequent activation of PKA. BafA1 has been used to assess autophagy flux, because it is reported to completely block the lysosomal degradation of LC3-II. cAMP has been reported to have effects on lysosomal pH by promoting the assembly of v-ATPase^48^. Surprisingly, however, we found that cilostazol and cAMP were able to overcome the effect of BafA1; hence, it is unlikely that these effects of cilostazol and cAMP are mediated by increases in v-ATPase assembly. Instead, our results suggest the possibility that alternate lysosomal proton-entry routes exist that may be recruited and/or activated by PKA. Another possibility is that inhibition of v-ATPase by BafA1 is not permanent, and was somehow reversed by cAMP/PKA. Further studies will be needed to clarify the molecular basis of this action.

Another novel finding is that the effect of cAMP/PKA on lysosomal pH is accompanied by and dependent on increases in cytosolic and/or lysosomal free zinc levels. We previously demonstrated that zinc ionophores such as clioquinol acidify lysosomes and increase autophagy flux in chloroquine-exposed cells. Interestingly, the cAMP/PKA effect on lysosomal pH shares the same mechanism as that of zinc ionophores. Although a large fraction of free zinc appears to localize in lysosomes, because TPEN chelates both cytosolic and lysosomal zinc, it remains unclear whether cytosolic or lysosomal zinc plays the main role in changing lysosomal pH. The development of a lysosome-specific zinc chelator may be needed to address this issue. Also of interest, a recent report by Golan showed that, in breast cancer cells, ZnT2 (Zn^2+^ transporter 2) binds v-ATPase and induces zinc influx as well as lysosomal acidification, another example where lysosomal zinc is associated with lysosomal pH^63^.

How and from where is zinc released by cAMP/PKA? In cultured astrocytes, oxidative stress releases zinc mainly from Mts, especially Mt3^53,54^. In the case of cAMP/PKA, Mt3 is also likely the main source of zinc, since cAMP failed to increase free zinc levels in Mt3-null astrocytes. cAMP also failed to reacidify lysosomes in the presence of BafA1, further supporting the idea that zinc is required. Mt3 is a CNS-enriched form of Mts that has been shown to modulate lysosomal properties, including pH^49^. One remaining question is whether cAMP/PKA acts directly or indirectly at Mt3 to release zinc. Because we are unable to identify a PKA-phosphorylation motif in Mt3, an indirect effect seems more likely. One possibility is that ROS mediates zinc release from Mt3, reflecting the fact that cAMP can increase oxidative stress in astrocytes^64^.

A more important question is whether cilostazol/cAMP/PKA would be effective in neurodegenerative disease models. In an Aβ-exposure model of AD, Aβ was found to affect lysosomal pH in astrocytes, and thereby reduced cathepsin D activity in lysosomes. As was the case in the BafA1 model, cilostazol successfully restored lysosomal acidic pH; it also blocked the Aβ inhibitory effect on cathepsin D activity. These observations indicate that cilostazol is effective in restoring defective lysosomal functions induced by Aβ. Consistent with this, cilostazol substantially reduced the accumulation of Aβ in astrocytes.

In the current study, we showed that activating the cAMP/PKA pathway, in this case with a PDE3 inhibitor, was effective in restoring the acidity of lysosomes. Although we used a PDE3 inhibitor to increase cAMP levels in the present study, it seems possible that other PDE inhibitors might have similar effects, depending on the PDE isoform expression profile in the cell type under investigation. While the precise mechanisms linking cAMP/PKA to Mt3 and zinc, and ultimately to lysosomal pH, require further elucidation, because a number of PDE inhibitors are being used safely in human patients, it may be worth examining their potential as therapeutics for various neurodegenerative conditions. One cautionary note is while our findings support the role of astrocytic Mt3 downregulation in accumulation of Aβ, the fact that Mt3 downregulation may reduce the potential boosting effect of cAMP on lysosomal functions, would diminish the potential relevance of cilostazol or other PDE inhibitors as a therapeutic measure in AD.

## Materials and Methods

### Chemicals

Cilostazol (Cilo), bafilomycin A1 (BafA1), and tetrakis(2-pyridylmethyl)ethylenediamine (TPEN) were obtained from Sigma (St. Louis, MO, USA). Fluorescein isothiocyanate (FITC)-labeled Aβ_1–42_ and soluble Aβ_1–42_ were obtained from the American Peptide Company (Sunnyvale, CA, USA).

### Astrocyte culture

Cortical astrocyte cultures were prepared from newborn mice as previously described^65–67^. Briefly, cerebral cortices were dissociated to produce single-cell preparations. The resulting cells were plated in Dulbecco’s modified Eagle’s medium (DMEM) supplemented with horse serum, 7% fetal bovine serum (FBS), and penicillin-streptomycin (100 IU/ml), and then incubated in a humidified 5% CO_2_ chamber at 37 °C. The media were changed every 3 days; to selectively grow adherent astrocytes, the plates were shaken prior to media change. Adherent astrocytes were cultured until confluence (2–4 weeks *in vitro*). Immunostaining for glial fibrillary acidic protein (GFAP) showed that the astrocyte cultures were of high purity (>95%). Except for FBS (Hyclone; Logan, UT, USA), the culture reagents were obtained from Invitrogen (Carlsbad, CA, USA).

Mt3-null (Mt3 KO) and Mt3 wild-type (Mt3 WT) mice were obtained by breeding among Mt3^+/−^ mice with C57B16/129sv hybrid background. Dr. R. D. Palmiter (University of Washington, Seattle, USA) kindly provided the Mt3-null mice. Genotyping with polymerase chain reaction (PCR) was carried out using the WT-specific sense primer 5′-CTC TCT ACA GAG GCC CGG CAG TCA C-3′ or the primer 5′-CAC AGT CCT TGG CAC ACT TCT CAC ATC CG-3′ (for both types). All mice were maintained at the dedicated animal facility at the Asan Institute for Life Sciences, Asan Medical Center, University of Ulsan College of Medicine.

All animal experiments were approved by the Institutional Animal Care and Use Committee (IACUC) of the Asan Institute for Life Sciences at Asan Medical Center. License Committee Approval Number is 2017-12-141 (Cortical Astrocyte cultures), 2016-12-288 and 2019-12-343 (Mt3 WT and KO mice cortical astrocyte cultures).

### Western blot analysis

Western blot analysis was carried out according to a previously described protocol^68^. Briefly, the cells were lysed in RIPA buffer (20 mM Tris-Cl pH 7.4, 1 mM EDTA, 150 mM NaCl, 1% Triton X-100, 1 mM EGTA, 1 μM Na_3_VO_4_, 2.5 mM sodium pyrophosphate, 1 mM phenylmethylsulfonyl fluoride, 1 μg/ml leupeptin,). The protein concentration was calculated using the BCA Protein Assay Reagent (BioRad, Hercules, CA, USA). Protein samples were separated by SDS-PAGE and transferred to polyvinylidene difluoride (PVDF) membranes (Millipore, Bedford, MA, USA). The membranes were incubated at 4 °C overnight with primary antibodies followed by horseradish peroxidase (HRP)-conjugated goat anti-rabbit IgG (1:5,000; Pierce; Carlsbad, CA, USA). The primary antibodies used were anti-6E10 (1:1000; Covance (Princeton, NJ, USA)), anti-mHttQ (1:1000; Cell Signaling (Danvers, MA, USA)), and anti-β-actin (1:5000; Sigma). All membranes were visualized using a UVP Autochemi Darkroom Imaging System (Utra-Violet Products Ltd. UK) and Immobilon Crescendo Western HRP Substrate or Luminata Forte Western HRP Substrate (Millipore, USA).

### Acridine orange staining

Cells were stained with acridine orange according to the manufacturer’s instructions (AO; Life Technologies (Carlsbad, CA, USA)) for 10 minutes, then washed twice in Dulbecco’s phosphate-buffered saline (DPBS). Lysosomes were visualized by monitoring red signals obtained using an excitation filter of 460 nm (450–480 nm) and a long-pass>515 nm emission/barrier filter.

### Live-cell confocal microscopy

Live-cell confocal microscopy was carried out according to a previously described protocol^67^. Briefly, astrocytes cultured on poly-L-lysine–coated coverslips were stained with 5 μM Zinpyr-1 (Mellitech, France) or 2.5 μM Fluozin-3-AM (Invitrogen) in minimum essential media (MEM) for 30 minutes in a humidified CO_2_ incubator and then transferred to Live Cell Imaging Solution (Molecular Probes; Carlsbad, CA, USA). A confocal imaging system (Carl Zeiss LSM 780, Zen software; Oberkochen, Germany, Leica TCS_SP2, Solmes, Germany) was used to obtain the resulting images.

### Clearance of GFP-tagged mutant huntingtin Q74 aggregates (GFP-mHttQ74) by cilostazol

According to a previously described protocol^67^, astrocytes were transfected for 6 hours with GFP-mHttQ74 using Lipofectamine 2000 (Invitrogen; Carlsbad, CA, USA). After washing with MEM, the cells were treated for 14 hours with the indicated drugs. During the 14-hour period after the drug exposure, the cells were observed under an Olympus IX70 fluorescence microscope (Olympus, Tokyo, Japan). Image J (NIH, USA) was used to count the number of mHttQ74 aggregates. The GFP-positive-mHttQ74 aggregate was quantified by dividing the number of GFP-positive-aggregate containing cells by the total number of GFP-positive cells. Then, the quantified values were graphed by percentage^67^.

### Fluorescence microscopic detection of changes in lysosomal pH

For detection of changes in lysosomal pH, astrocytes cultured on poly-L-lysine–coated glass slides were stained with 5 μM Lysosensor DND-189 or Lysosensor Yellow-Blue DND-160 (Invitrogen) dye in growth medium for 30 minutes in a humidified CO_2_ incubator. The cells were then transferred to Live Cell Imaging Solution and the resulting images were obtained using an LSM780 confocal Live-Cell Imaging System.

### Statistical analysis

All results are presented as means ± SEM. Two-tailed Student’s t-test was used to evaluate the significance of differences between groups. Two-tailed Student’s t-test with Bonferroni correction or one-way ANOVA test with post-hoc Fisher exact test was used for multiple comparisons. Two-tailed Student’s t-test statistical analyses and graphical presentations were conducted and created using Sigma Plot version 10.0 software. One-way ANOVA test with post-hoc Fisher exact test statistical analyses and graphical presentations were conducted and created using Prism version 5.01 software.

All experimental protocols were approved by Asan Institute for Life Sciences at Asan Medical Center and Ulsan University College of Medicine and all methods were performed in accordance with the relevant guidelines and regulations.

## Supplementary information

Supplementary data.
