# Supplementary material for: Cilostazol restores autophagy flux in bafilomycin A1-treated, cultured cortical astrocytes through lysosomal reacidification: roles of PKA, zinc and metallothionein 3
Source: Sci Rep. 2020 Jun 8;10:9175. doi: 10.1038/s41598-020-66292-3 (PMC7280249; doi:10.1038/s41598-020-66292-3)
Supplement: Supplementary file 1 — Supplementary data. [file 41598_2020_66292_MOESM1_ESM.pdf]

Cilostazol restores autophagy flux in bafilomycin A1-treated, cultured cortical astrocytes through lysosomal reacidification: roles of PKA, zinc and metallothionein 3

Ha Na Kim[2], Bo-Ra Seo[2], Hyunjin Kim[1] and Jae-Young Koh[1,2*]

[1] Department of Neurology, University of Ulsan College of Medicine, Seoul, Korea; Department of Neurology, Asan Medical Center, University of Ulsan College of Medicine, Seoul, Korea

[2] Neural Injury Lab, Biomedical Research Center, Asan Institute for Life Sciences, Asan Medical Center, Seoul, Korea

***To whom correspondence should be addressed:** Jae-Young Koh

Department of Neurology, Asan Medical Center, University of Ulsan College of Medicine, 88, Olympic-ro 43-gil, Songpa-gu, Seoul 05505, Republic of Korea

Phone: 82-2-3010-4127; Fax: 82-2-483-5446; E-mail: jkko@amc.seoul.kr

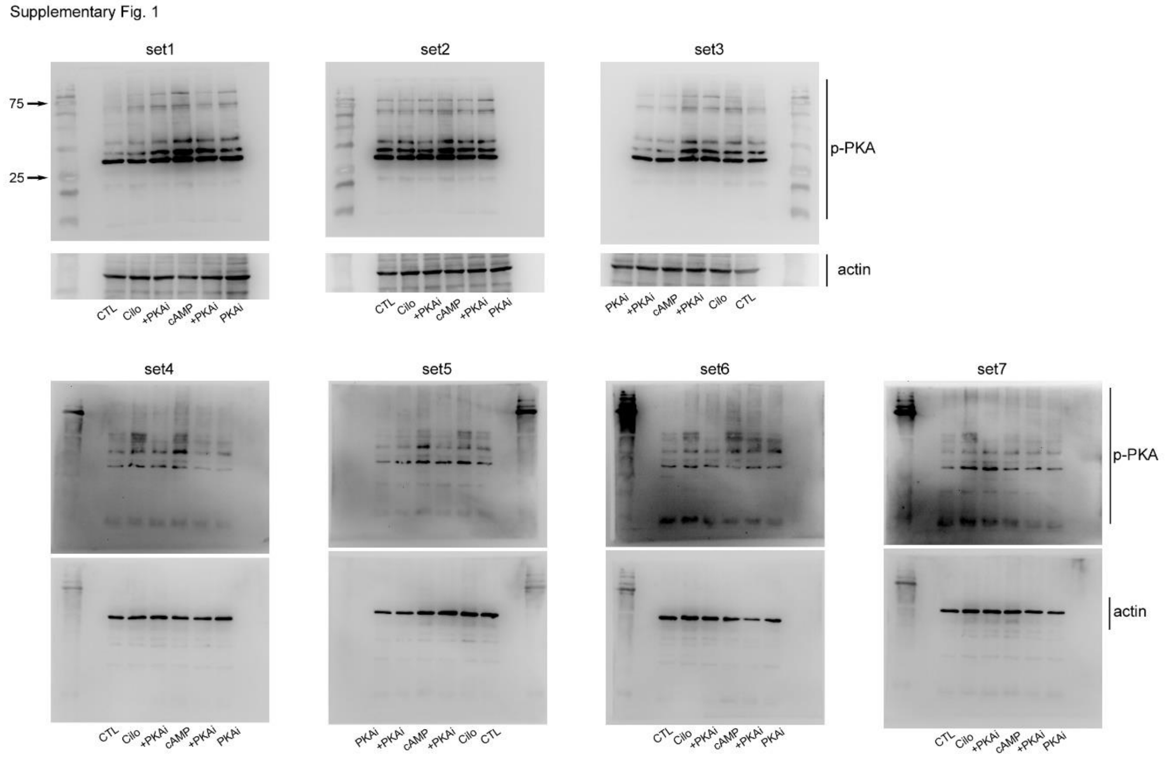

Supplementary Fig. 1 PKA may mediate the effects of cilostazol and cAMP.

Original blot of western blots for p-PKA in cells treated with 10 μM cilostazol (+Cilo) or 300 μM cAMP alone (+cAMP), or together with 10 μM H-89 (+PKAi), a PKA inhibitor, for 1 hour.

Supplementary Fig. 2

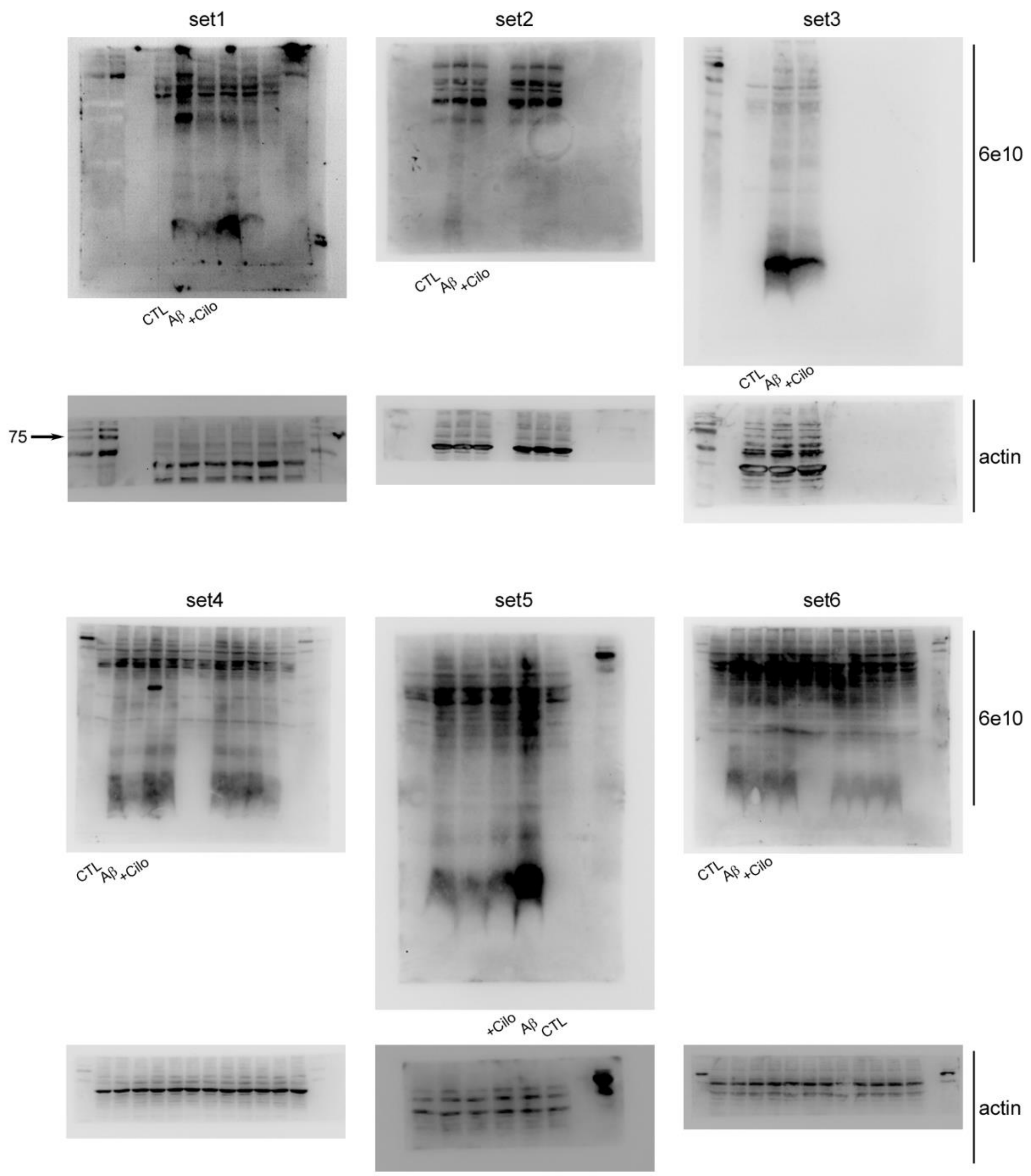

Supplementary Fig. 2 Cilostazol reduces accumulation of Aβ and huntingtin aggregates in astrocytes.

Original blot of western blots analyses of lysates from cells that were sham-washed (CTL), or exposed for 24 hours to Aβ alone or Aβ plus cilostazol (+Cilo). Levels of Aβ monomers, tetramers, and oligomers were decreased by Aβ plus cilostazol compared with Aβ alone.

Supplementary Fig. 3

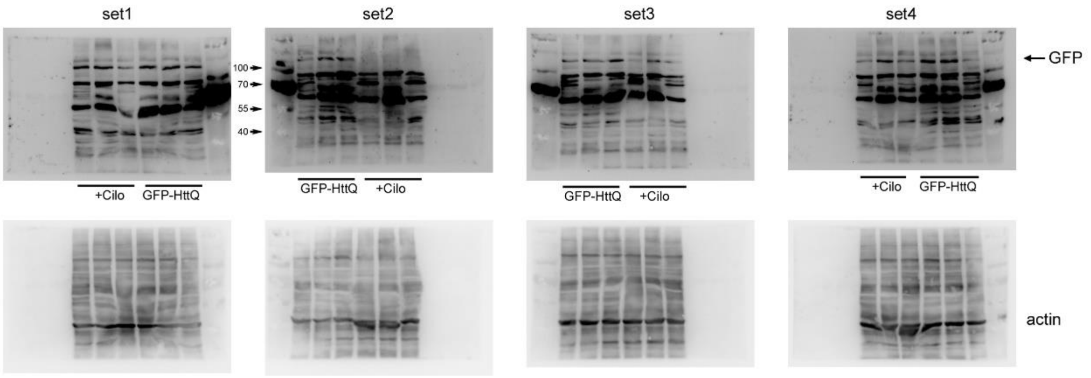

Supplementary Fig. 3 Cilostazol reduces accumulation of Aβ and huntingtin aggregates in astrocytes.

Original blot of western blots analysis of GFP aggregates obtained from GFP-mHttQ74–overexpressing astrocytes following cilostazol treatment. Transfected cells were sham-washed (mHttQ) or treated with 10 μM cilostazol (+Cilo) for 14 hours.

Supplementary Fig. 4

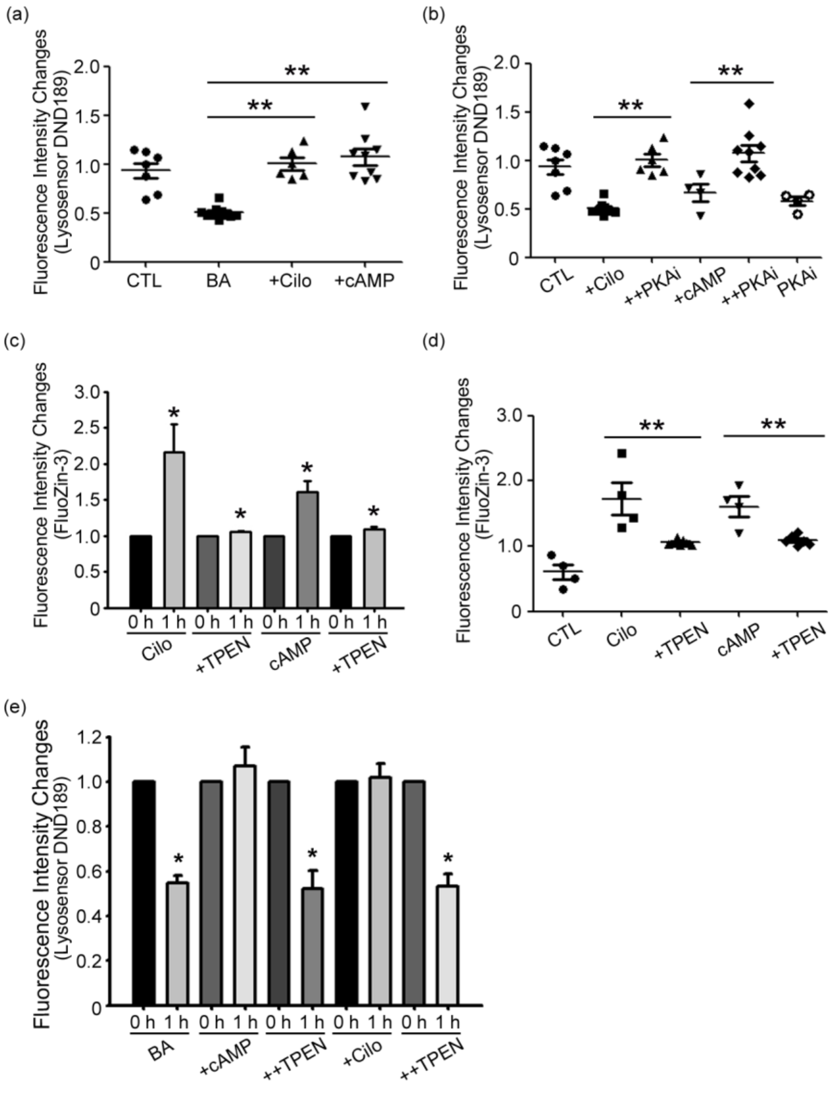

Supplementary Fig. 4 Quantification data

(a) Data indicate relative changes in DND-189 fluorescence intensity. Values were normalized to control values (mean ± SEM; n = 6; ** denote $P < 0.01$ compared with BA, Cilo, or cAMP; one-way ANOVA with post-hoc Fisher exact test for 2 comparisons).

(b) Data indicate relative changes in the DND-189 fluorescence intensity. Values were normalized to control values (mean ± SEM; n = 4; ** denote $P < 0.01$ compared with BA, Cilo, cAMP, or PKAi; one-way ANOVA with post-hoc Fisher exact test for 2

comparisons).

(c) Bars indicate relative changes in the fluorescence intensity normalized to control values (mean ± SEM; n = 4; * denote $P < 0.05$ compared with BA, Cilo, cAMP or TPEN; Two-tailed Student's t-test for 2 comparisons).

(d) Relative changes in the FluoZin-3 fluorescence intensity normalized to control values (mean ± SEM; n = 4; ** denote $P < 0.01$ compared with BA, Cilo, cAMP or TPEN; one-way ANOVA with post-hoc Fisher exact test for 2 comparisons).

(e) Relative changes in the DND-189 fluorescence intensity normalized to control values (mean ± SEM; n = 4; * denote $P < 0.01$ compared with BA, Cilo, cAMP or TPEN; Two-tailed Student's t-test for 2 comparisons).

Supplementary Fig. 5

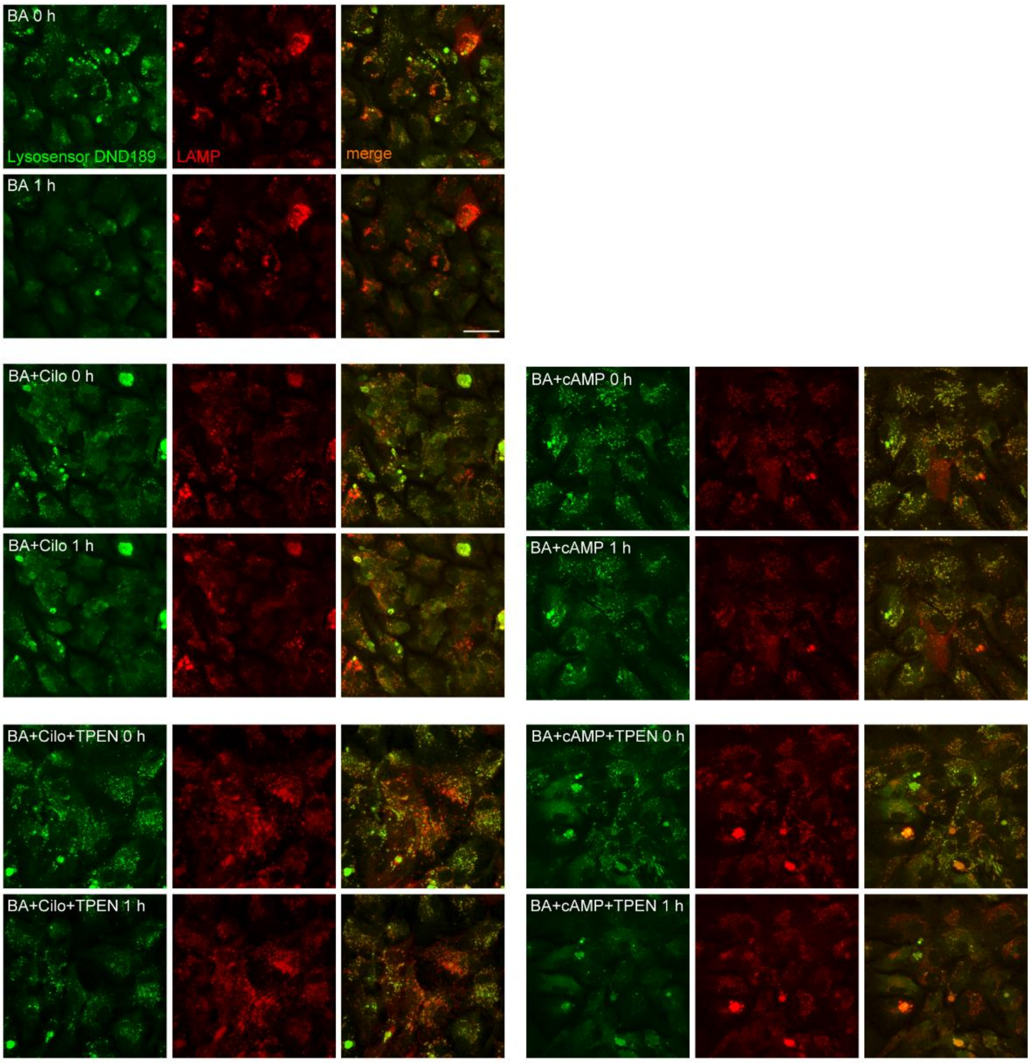

Supplementary Fig. 5 Identification of lysosomes using LAMP in Figure 3

(a) Changes in intracellular and lysosomal free zinc levels were visualized by loading astrocytes with FluoZin3-AM for 30 minutes, followed by a sham wash (CTL) or treatment for 60 minutes with 10 µM cilostazol or 300 µM cAMP (n = 3). LAMP1-positive particles exhibited red fluorescence in resting astrocytes (CTL). Cilostazol and cAMP further increased free zinc levels, most of which colocalized with LAMP1 fluorescence (Scale bar, 20 µm).
